# Atlanta Quackery

**Published:** 1874-12

**Authors:** 


We are constantly receiving letters from physicians and others, making inquiries in regard to various institutions, associations and individuals in this city and vicinity, who are flooding the country with their nauseous literature, in the form of posters, pamphlets and newspaper puffs. These are usually strikingly illustrated with wood-cuts, setting forth the wonderful and miraculous cures, attested, as a general thing, by numerous certificates, on the order of Helmbold's buchu and other nostrum vendors in this once free and happy country.

Our medical friends who desire information have not seen, we feel confident, a specimen of their flourishing posters or illustrated pamphlets. We advise them to procure at once a specimen, which they can readily do in almost any locality, when they will find prominent in all of them the "ear-marks" of the charlatan; that class of gentry who are ever ready to speculate on the misfortunes of poor, suffering humanity; thus prostituting the noble calling of the physician for the paltry sum of a few dollars.

There should not be a member of our profession who does not know that such publications never emanate from a member of the profession in good standing, but that they are, whether from institutes, associations or individuals, effusions of medical charlatans, or degenerate disciples of the healing art.

---

CRITICAL ILLNESS in the family of Dr. Battey has delayed the issue of the current number of the JOURNAL.

---

IT IS SAD TO LEARN that the differences among the members of the State Medical Association of Arkansas have assumed so serious an aspect as to culminate in a published call for a meeting of the opposition, with the avowed object of organizing a rival State Association.

